# Physico-chemical characteristics and aflatoxins production of *Atractylodis Rhizoma* to different storage temperatures and humidities

**DOI:** 10.1186/s13568-021-01316-3

**Published:** 2021-11-25

**Authors:** Qiutao Liu, Lingling Jiang, Lihe Xiao, Weijun Kong

**Affiliations:** 1 NMPA Key Laboratory for Quality Research and Evaluation of Traditional Chinese Medicine, Shenzhen Institute for Drug Control, 518057 Shenzhen, PR China; 2 Institute of Medicinal Plant Development, Chinese Academy of Medical Sciences and Peking Union Medical College, 100193 Beijing, PR China

**Keywords:** *Atractylodis rhizoma*, Aflatoxin, Storage, Physico-chemical characteristics, Central composite design-response surface methodology

## Abstract

This study aimed to investigate the characteristics, moisture contents, chemical fingerprints changes and aflatoxins accumulation of *Atractylodis rhizoma* during storage, further to determine the optimum temperature and relative humidity conditions. Based on the suitable temperature (20–40 °C) and relative humidity (80–95%), 13 different temperature and humidity conditions were set up by the central composite design-response surface methodology (CCD-RSM) for *Aspergillus flavus*. After inoculation with *Aspergillus flavus* by artificial infection, *A. rhizoma* samples were stored under normal conditions and 13 different temperature and relative humidity levels. By taking the changes of characteristics, the contents of moisture, chemical fingerprints and aflatoxins as the evaluation indexes for *A. rhizoma* with or without *Aspergillus flavus* fungi to optimize the optimal storage conditions. After storage for 10 days, the color of *A. rhizoma* was deepened, the water content and chemical composition increased, and some unknown components were detected. The susceptible condition for aflatoxins production in *A. rhizoma* was identified at temperature 22–37 °C and relative humidity over 87.5%. Thus, the suitable storage conditions for *A. rhizoma* should be controlled at temperature below 20 °C and relative humidity less than 85%. This paper screened out the optimum temperature and humidity for the storage of *A. rhizoma*. Then, the storage specification for *A. rhizoma* was proposed, lying technical and data support for the scientific preservation of other food or herbs.

**Supplementary Information:**

The online version contains supplementary material available at 10.1186/s13568-021-01316-3.

## Introduction

The quality and safety of foods and traditional Chinese medicines (TCMs) are extremely important for their clinical application, and scientific storage to prevent mildew and mycotoxins contamination is the basis and premise. China has a vast territory and a large span from the north to the south, especially in the south of China, it is warm and humid all the year round. In the rainy season, especially in June and July each year, the air humidity and atmospheric temperature are very high, which are suitable for the growth of most of the toxicogenic fungi in TCMs, leading to many difficulties for their safe storage. In addition, most TCMs are rich in polysaccharides, protein, starch, fat oil and other components, which provide good nutritional conditions for the rapid reproduction and growth of mould including toxicogenic fungi, followed by the mycotoxins contamination (Christensen et al. 2012; Huang et al. 2019; Zhang et al. 2018). Mycotoxins as secondary metabolites produced by many kinds of fungi when the climatic conditions become favorable during the growth, harvest, handling, especially the storage processes of TCMs (Weaver et al. 2020; Liu et al. 2015; Wang et al. 2015; Duarte et al. 2020). Various mycotoxins with strong toxigenic, carcinogenic, mutagenic and immunosuppression properties and serious threats to human health have been found in diverse TCMs all over the world in the past decades (Yang et al. 2020; Ponzilacqua et al. 2019; Nieto et al. 2018). High occurrence of mycotoxins in TCMs, along with their toxic effects, as well as huge economic losses to TCMs industries have resulted in a global concern (Haque et al. 2020; Ashiq et al. 2014). Aflatoxins (AFs) produced by *Aspergillus* strains, due to their strong liver and kidney toxicity and high incidence in many food and agricultural products, and TCM matrices, have attracted much attention (Ali 2019; Martins et al. 2020). They have been listed as the most dangerous food hazards in nature by the Food and Agriculture Organization (FAO) of the United Nations and the World Health Organization (WHO) (World Health Organization 2007–2015). Of them, AFB_1_ has strong hepatotoxic and hepatocarcinogenic properties. The International Agency for Research on Cancer has classified AFB_1_ as Group 1A carcinogen (International Agency for Research on Cancer [IARC] 1999).

*Atractylodis rhizoma*, a classical TCM, is the dry rhizoma of *Atractylodes lancea* (Thunb.) DC. (*A. lancea*) and *Atractylodes chinensis* (DC.) Koidz. (*A. chinensis*) and exhibits in 2020th edition of the Chinese Pharmacopeia (Chinese Pharmacopoeia Commission 2020). In addition, *A. rhizoma* has been prescribed in Chinese, Korean, Japanese and Thai traditional medicine in present paper (Zhang et al. 2021). *A. rhizoma* is an important crude drug that commonly believed to be used in the treatment of influenza, night blindness, rheumatic dis-eases and several other types of digestive problem. (Kitajima et al. 2003; Jin et al. 2004). Unfortunately, a large amount of starch and volatile components contained in *A. rhizoma*, in combination of favorable environmental conditions make it easily subject to fungal contamination and mycotoxins residue (Liu et al. 2016a, b). Our previous studies have found that 4 out of 22 batches of *A. rhizoma* samples contaminated trace levels of mycotoxins (Liu et al. 2019; Hu et al. 2015). High incidence of mycotoxins in *A. rhizoma* will pose potential threats and influence on the quality and safety of this TCM, which is great importance and necessity, however, has not been clarified from then on.

Therefore, this study aimed to first investigate the toxigenic fungi growth and aflatoxins production in *A. rhizoma* under various humidity and temperature conditions according to the central composite design-response surface methodology (CCD-RSM) (Kumar et al. 2016; Duangjit et al. 2015; Norioko et al. 2013), then to explore the relationship of mycotoxins accumulation determined by using an ultra-fast liquid chromatography/tandem mass spectrometry (UFLC-MS/MS) method and bioactive components variation measured by introducing a gas chromatography with flame ionization detection (GC-FID) technique, and naturally to elucidate fungal contamination and mycotoxins production affected the internal and external quality of this TCM for screening for the most suitable temperature and humidity conditions for safe storage of *A. rhizoma*.

## Materials and methods

### Chemicals and standards

Dried *A. rhizoma* samples were purchased from the Anguo medicine market in Hebei province, China. All the samples were crushed into powder and passed through a third-sieve, and then stored at − 20 °C until further use.

The lyophilized powder of aflatoxigenic *Aspergillus flavus* (CGMCC 3.4410) was supplied by the China General Microbiological Culture Collection Center (CGMCC, Beijing, China). *Aspergillus flavus* dissolved in 0.5 mL of sterile water (121 °C, 20 min) for culture on Salt Czapek Dox Agar medium (Beijing Aobox Biotechnology Co., Ltd., Beijing, China) at constant temperature and humidity (28 °C, 75% relative humidity, RH) under a 12/12 h daylight/dark regime.

Chromatographic-grade methanol, acetonitrile and ethyl acetate were supplied by Fisher Scientific (Fair Lawn, NJ, USA). Tween-20 and formic acid were supplied by Xilong Chemical Corporation (Guangdong, China). Magnesium sulfate anhydrous (MgSO_4_) were provided by Sinopharm Chemical Reagent Co., Ltd. (Shanghai, China). Primary secondary amine (PSA) sorbent was purchased from Agela Technologies (Tianjin, China). The water used throughout the experiment was Wahaha purified water (Hangzhou, China).

**Fig. 1:**
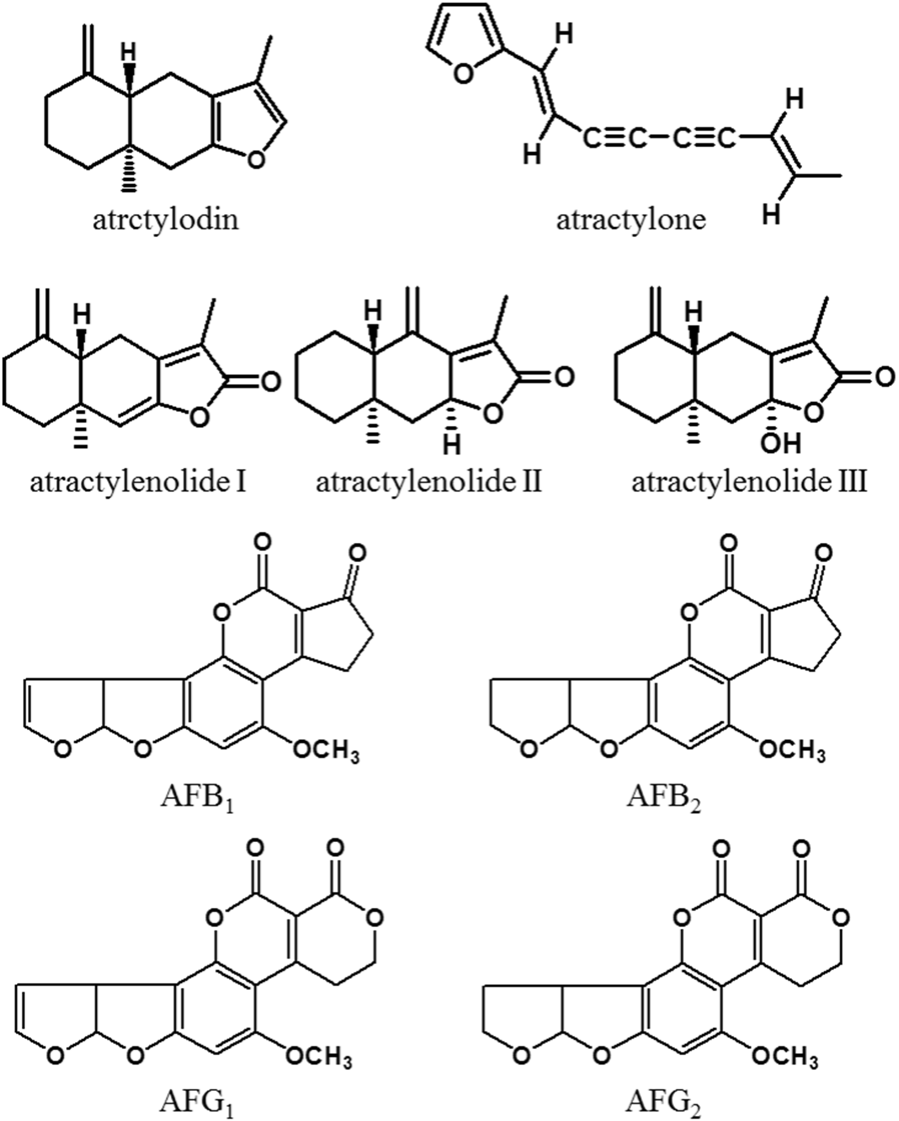
Chemical structures of 5 chemical components in *A. rhizoma* and 4 aflatoxins

The stock solution containing four aflatoxins (AFB_1_, AFB_2_, AFG_1_ and AFG_2_, the chemical structures shown in Fig. 1) was stored in methanol and supplied by SUPELCO (Bellafonte, PA, USA). The reference standards (atractylon, atractydin, atractylenolide I, II and III) were provided by Chengdu Chroma Biotechnology (Chengdu, China) with purity higher than 98.0% and chemical structures in Fig. 1. *n*-Tridecane as the internal standard was purchased from Dr. Ehrenstorfer (Augsburg, Germany) with purity over 99%.

### Preparation of *Aspergillus flavus* spore suspension

Spores of *Aspergillus flavus* CGMCC 3.4410 were propagated on Salt Czapek Dox Agar (SCDA) medium for 14 days at 28 ^o^C. Spore culture on SCDA medium from the fungal were collected by suspension in 10 mL water containing 0.1% tween-20. The suspension formed was filtered through 3 layers of cheesecloth. A final spore solutions containing 10^6^ spores/mL was adjusted by sterile water using a haemocytometer (Obata et al. 2019). The inoculation and sporangia images were shown in Additional file 1: Fig. S1.

### Sample inoculation with *Aspergillus flavus* spore suspension

The suitable temperature and humidity for the growth of *Aspergillus flavus* are 25–40 °C and 80–90%, respectively. The optimal conditions predicted by the Design-Expert V8.0.6 software. Design of experiment analysis utilizes a CCD-RSM to optimize the suitable temperature and humidity for storage of *A. rhizoma* samples. Create a temperature and humidity chamber in a MJX Intelligent mould culture box (Ningbo Jiangnan Instrument Factory, China).

Surface sterilized culture dishes were irradiated under an UV lamp for 1 h, while the samples were sterilized by irradiation both sides for 30 min by 265 nm ultraviolet rays. 50 g of *A. rhizoma* sample was placed to each dish. 1 mL of spore suspensions was inoculated onto the surface of each sample for culturing under a 12 h day/night photoperiod under 13 different temperature and humidity conditions for 10 days. After the culture period, the characteristics was observed and the contents of moisture, chemical fingerprints changes and aflatoxins accumulation of *A. rhizoma* were investigated to determine the optimum temperature and relative humidity conditions.

### Establishment of UFLC-MS/MS method for determination of aflatoxins

#### Equipment and UFLC-MS/MS conditions

The UFLC-MS/MS equipment consisted of Shimadzu ultra-fast liquid chromatography (UFLC) system (Shimadzu, Kyoto, Japan) coupled to an AB SCIEX QTrap® 5500 mass spectrometer (AB SCIEX, Foster City, CA, USA) with electrospray ionization (ESI) source. The chromatographic separation was carried out on a SHISEIDO CAPCELL CORE C_18_ column (50 mm × 2.1 mm, 2.7 μm, Shiseido). A binary (acetonitrile with 0.1% formic acid (A) and 0.1% (*v/v*) aqueous formic acid (B)) solvent system was optimized for analysis: 0–2 min, 90–80% B; 2–8 min, 80–30% B; 8–10 min, 30–5% B; 10–12 min, held at 5% B; 12.01–15 min, 90% B. The flow rate was set at 0.3 mL/min and the injection volume was 2 µL.

Quantification was conducted with positive electrospray ionization (ESI^+^). The ion spray (IS) voltage was 5500 V and the source temperature was 550 °C. Nebulizer gas (GS1), heater gas (GS2) and curtain gas (CUR) were ultrahigh purity nitrogen and the values were adjusted to 50, 50 and 35 psi, respectively, as well as the collision activation dissociation gas (CAD) was medium. The precursor-to-product ion for AFB_1_, AFB_2_, AFG_1_ and AFG_2_, and the MS/MS parameters including collision energy (CE), collision cell exit potentials (CXP), entrance potential (EP) and declustering potential (DP) were shown in Table 1. The acquired data was processed with Analyst® software version 1.6.2 (AB SCIEX, Foster City, CA, USA).

**Table 1:** MS/MS parameters of AFG_2_, AFG_1_, AFB_2_ and AFB_1_

Mycotoxin	Precursor ion	Product ion	Dp (V)	CE (V)	CXP (V)	Dwell time (ms)
AFG_2_	331.1	245.1 (Q)	130	40	17	80
		217.0 (q)		48	15	
AFG_1_	329.0	243.1 (Q)	130	37	13	80
		215.0 (q)		44	14	
AFB_2_	315.0	287.1 (Q)	170	36	16	80
		259.1 (q)		40	16	
AFB_1_	313.0	285.1 (Q)	110	32	17	80
		269.0 (q)		43	17	

*Q* quantification ion, *q* qualitative ion, *DP* declustering potential, *CE* collision energy, *CXP* collision cell exit potential

#### Preparation of sample solution

Extraction of aflatoxins from *A. rhizoma* sample was performed by modified QuEChERS (Quick, Easy, Cheap, Effective, Rugged and Safe) procedure. 1 g *A. rhizoma* powder was accurately weighted into a 30-mL tube, with the addition of 4 mL of 90% aqueous acetonitrile solution, followed by properly vortexing for 1 min and sonication for 5 min in an ultrasonic bath. Afterwards, 180 mg MgSO_4_ and 60 mg PSA were added, each tube was shaken with a vortex for 1 min and centrifuged for 10 min at 10,000 rpm. After that, 1 mL of the upper organic phase was transferred into a 2-mL centrifugation tube, and concentrated to dryness under a stream of nitrogen at 40 °C. The extracts were reconstituted in 0.5 mL of 50% aqueous methanol solution. Subsequently, the extract solution was shaken vigorously for 30 s and filtered through a 0.22-µm PTEE filter and transferred to an autosampler vial for analysis by the UFLC-MS/MS system.

#### Matrix-matched calibration curve

Considering matrix effect in aflatoxins determination (Liu et al. 2019), matrix-matched calibration curves were used throughout this study by spiking blank matrix of the extracts of analyte-free sample with aflatoxin standards. Blank matrix was obtained by the preparation of sample as described. The calibration curves were prepared in a blank matrix spiked with a series of concentration at 1, 2, 5, 10, 25, 50 and 100 ng/mL of AFB_1_ and AFG_1_, 0.25, 0.5, 1.25, 2.5, 6.25, 12.5 and 25 ng/mL of AFB_2_ and AFG_2_ by plotting the peak areas (*y*) of each analyte against the concentration (*x*).

### Establishment of GC-FID fingerprint

#### Equipment and GC-FID conditions

GC analyses were carried out with an Agilent 6890N gas chromatograph coupled with a flame ionization detector (Agilent Technologies, Little Falls, DE, USA) and an Agilent 7683 autosampler. Data were collected and processed by a HP ChemStation (Hewlett Packard, Palo Alto, CA, USA). The chromatographic separation was performed on a HP-5 fused silica capillary column of 30 × 0.32 m and 0.25 μm film thickness. High-purity nitrogen (> 99.99% purity) was used as carrier gas. The FID detector was fed by hydrogen (40 mL/min) and air (450 mL/min). The injection volume of sample was 2 µL. The injector and detector temperatures were both 250 °C, and the injection was performed in the splitless mode with a purge time of 0.75 min. The temperature program for GC was as follows: initial temperature 80 °C held for 2 min, increased at rate of 10 °C/min to 120 °C, second increased at 2 °C/min to 150 °C, third increased at 4 °C/min to 170 °C, and finally increased at 10 °C/min to 240 °C and held for 2 min, for a total runtime of 35 min. The content of each component in essential oil was comparing by their areas to the peak areas of IS, expressed as mean ± SD according to relative peak areas.

#### Preparation of standard solution

Appropriate amount of atractylon, atractydin, atractylenolide I, II and III was weighed accurately, respectively, with the addition of ethyl acetate to prepare a standard solution at the concentration about 1 mg/mL, which were used as qualitative control solutions.

#### Extraction of essential oils and preparation of sample solution

Essential oil from *A. rhizoma* sample was obtained using conventional hydrodistillation technique. 30 g *A. rhizoma* powder was accurately weighed into a flask, followed by the addition of 300 mL of distilled water for mixing evenly. Then, the solution was slowly heated to boiling and kept slightly boiling for about 5 h using specific instrumentation until the content of essential oils in the extractor did not increase. The essential oil was transferred to a close bottle, with the addition of anhydrous sodium sulfate to absorb water, followed by storage at low temperature in the dark. 50 µL of essential oil was accurately transferred to a 10-mL volumetric flask, with the addition of ethyl acetate, as well as *n*-tridecane as the internal standard (IS). The final concentrations were 1 µL/mL for IS and 5 µL/mL for sample solution.

## Results

### Characteristics

**Fig. 2:**
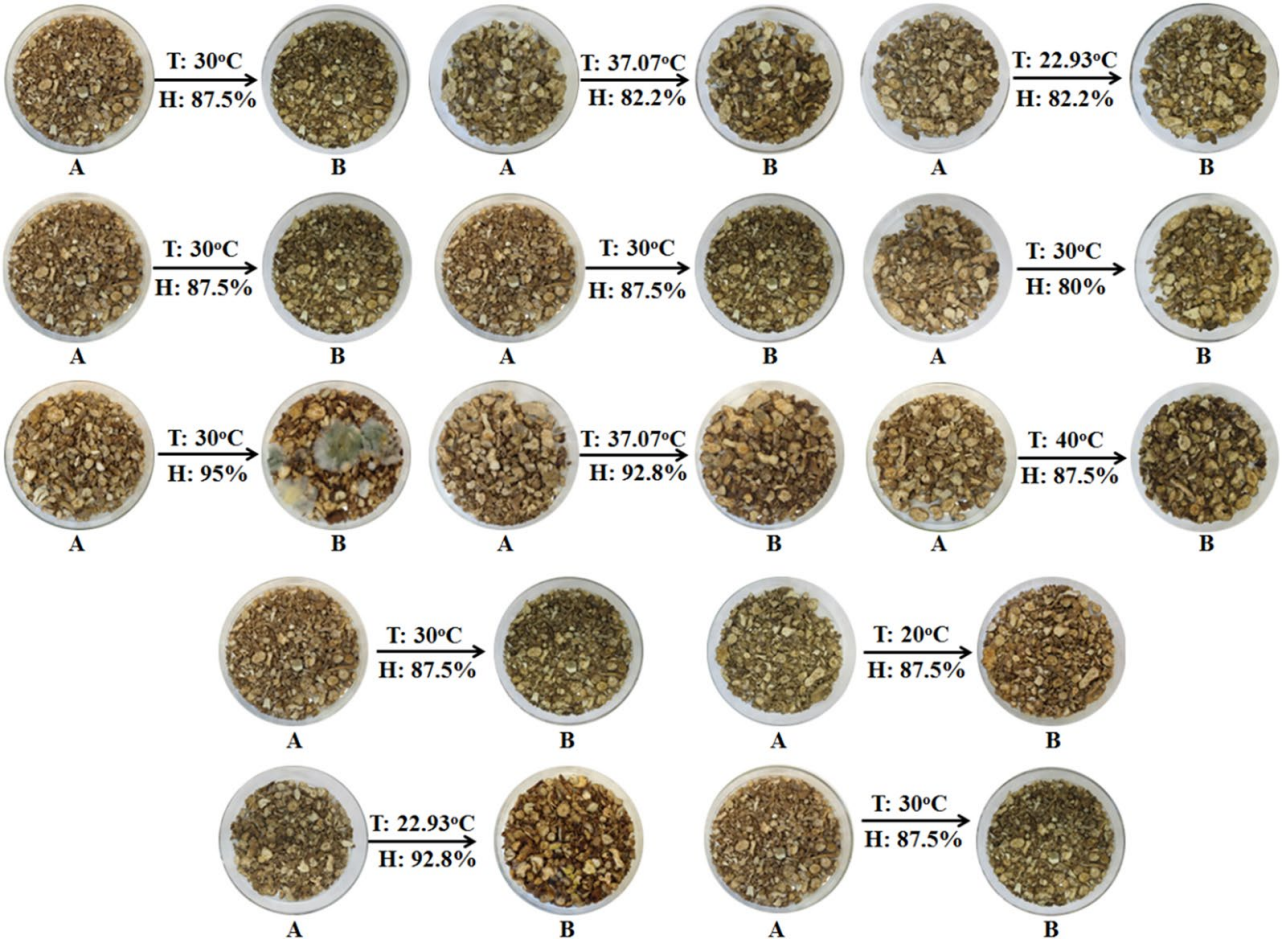
* A. rhizoma* samples stored at different temperature and humidity conditions for **A** 0 and **B** 10 days

Following storage for a long incubation period, as shown in Fig. 2, the color of *A. rhizoma* was gradually deepened. In the certain scope, the majority of *Aspergillus flavus* increased along with the temperature and humidity change. Fungi appeared on parts of *A. rhizoma* samples and were clearly visible. Especially, the samples were mildewed most seriously under temperature 30 °C and humidity 95%.

### Contents of moisture

Considering more volatile components in the *A. rhizoma* samples, toluene method was used to determine the water content and calculate the moisture content (%). The moisture content of *A. rhizoma* was not more than 11% according to Chinese Pharmacopoeia (2020th Edition). Table 2 showed that the set temperature and humidity have obvious influence on the moisture content, which was higher than the required limit, indicating that the sorption and hygroscopicity were serious when storage under different temperature and humidity. Also, the suitable environment provided convenience for the growth of *Aspergillus flavus*.

**Table 2:** Contents of moisture and AF(G_1_+ G_2_+ B_1_+B_2_) in *A. rhizoma* after incubation with *Aspergillus flavus* for storage at different temperature and humidity conditions

No.	Before /after storage	Factor A: Temp (^o^C)	Factor B: Hum (%)	Moisture	Content of AF (G_1_+G_2_+B_1_+B_2_) (µg/kg)
1	Before	Normal	Normal	10.98% ± 0.91%	–^a^
2	After	20.00	87.50	21.30% ± 0.62%	–
3	After	30.00	87.50	22.40% ± 0.88%	–
4	After	40.00	87.50	23.94% ± 0.32%	–
5	After	37.07	92.80	23.01% ± 0.26%	3.84
6	After	30.00	87.50	22.40% ± 0.56%	–
7	After	30.00	87.50	22.40% ± 0.50%	–
8	After	30.00	95.00	33.72% ± 0.33%	10.26
9	After	30.00	87.50	22.40% ± 0.87%	–
10	After	30.00	80.00	20.18% ± 0.66%	–
11	After	22.93	82.20	19.94% ± 0.61%	–
12	After	22.93	92.80	33.60% ± 0.53%	–
13	After	30.00	87.50	12.98% ± 0.34%	–
14	After	37.07	82.20	22.31% ± 0.90%	–

^a^ Not detected

**Fig. 3:**
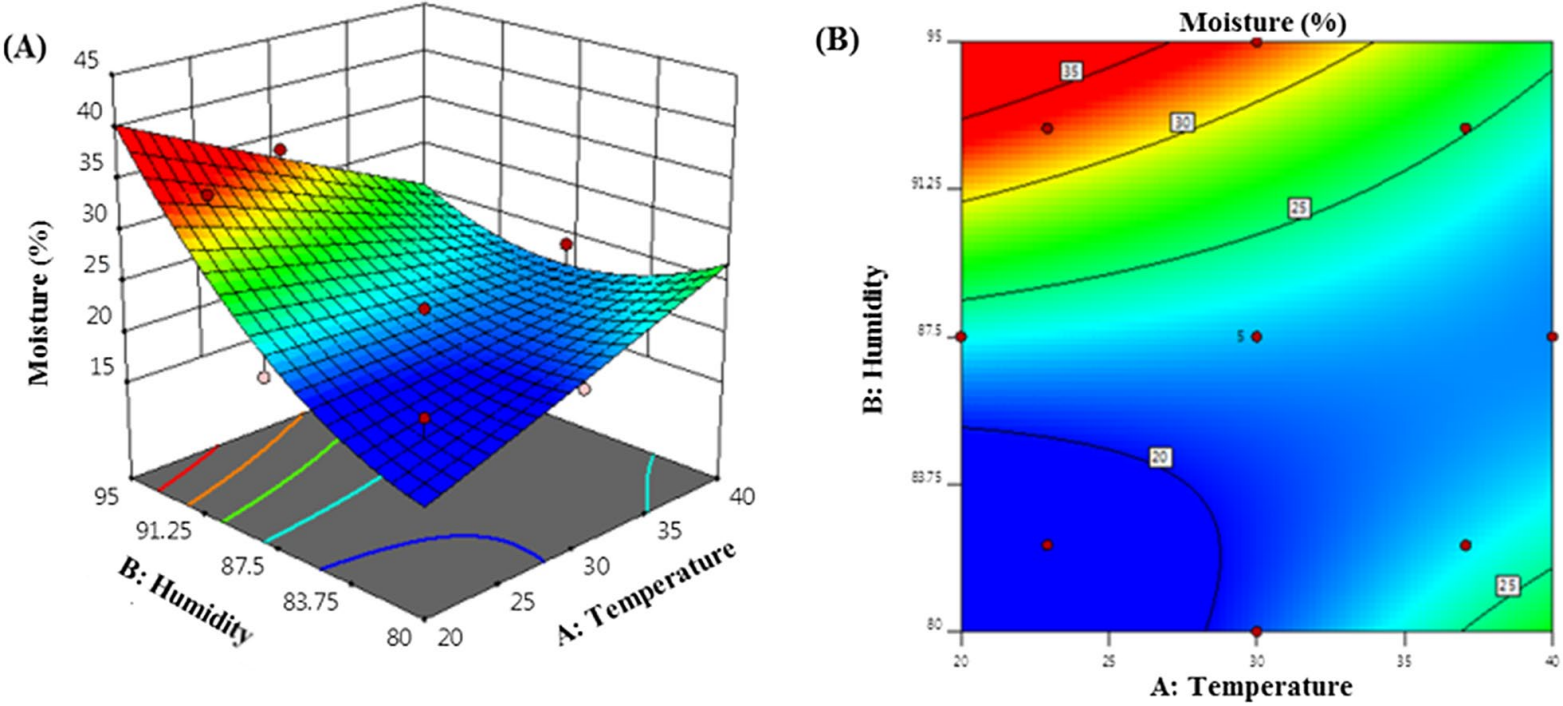
Response surface model for moisture

Further, response surface model was used to analyze the changing trend of moisture content under different temperature and humidity conditions (Fig. 3). When the temperature was set at 20 °C and humidity at 80%, the moisture content was the lowest, while at temperature 40 °C and humidity 95%, the moisture content was the highest.

### Component changes in chemical fingerprints

**Fig. 4:**
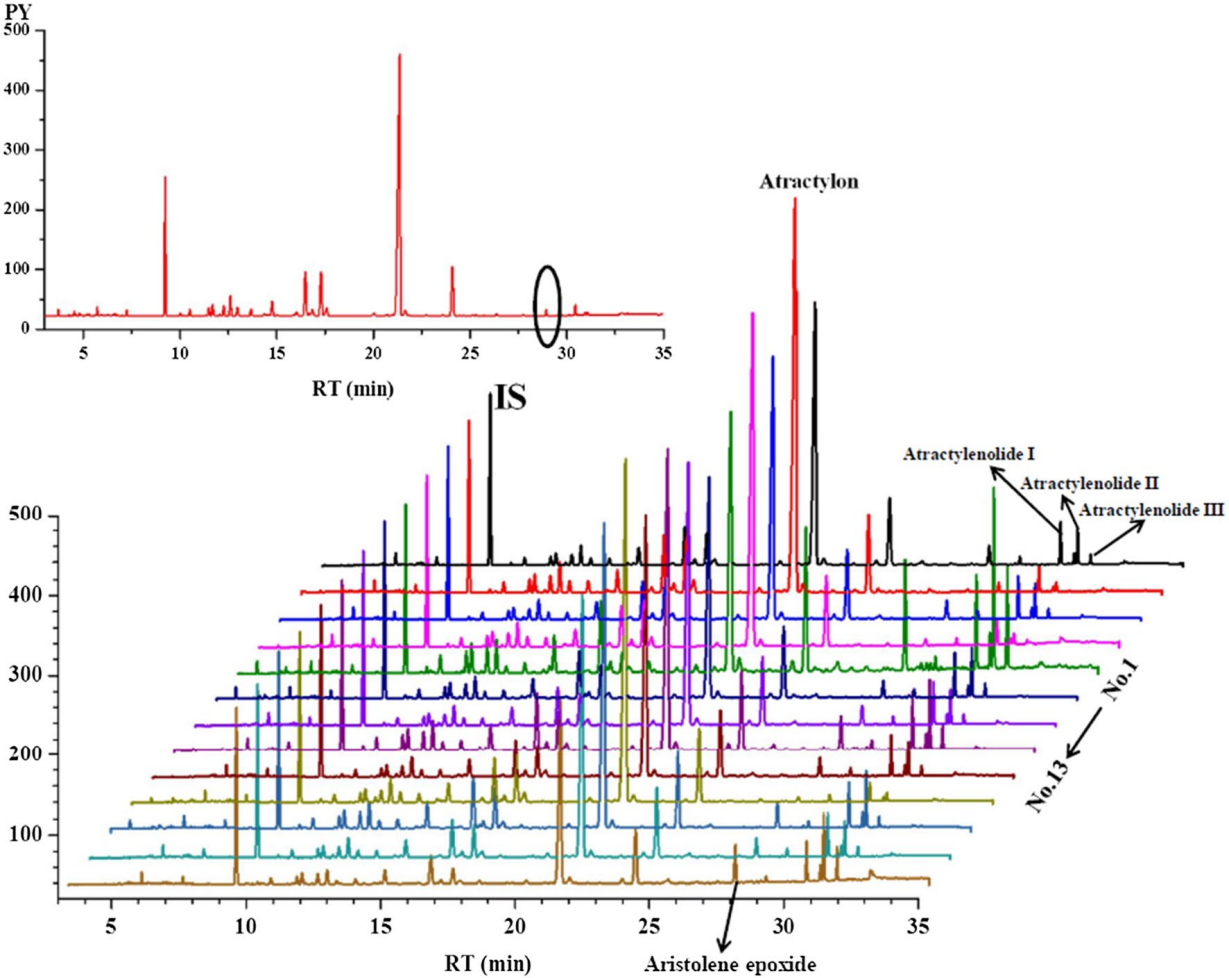
GC-FID chromatograms of *A. rhizoma* essential oil before and after storage at different temperature and humidity conditions

Essential oils from 14 batches of *A. rhizoma* samples extracted by hydrodistillation were analyzed by GC-FID. The GC-FID chromatograms presented all of the volatile components as chromatographic peaks. The chemical fingerprint of volatile oil was established. By taking the ratio of relative retention time (RRT) and relative peak area (RPA) of the each chromatographic peak to *n*-Tridecane (IS) as the index, the component changes were analyzed in Fig. 4.

First, the percentage of the total volume (mL) of extracted essential oil to the mass (g) of sample powder is considered as the yield (%) of essential oil. Yields in the *A. rhizoma* sample inoculated with *Aspergillus flavus* and stored under different temperature and humidity conditions were obviously reduced. Rise of temperature may accelerate the loss of volatile components and *Aspergillus flavus* may decompose the active component. Secondly, the higher the temperature was, the more complex chemical composition changes with suitable humidity were observer. The contents of atractylone, atractylenolide I, II, and III, especially at 37.07 °C and 92.8% humidity, changed most obviously (Additional file 1: Fig. S2). After a 10-day inoculation with *Aspergillus flavus*, a new chemical component was measured and identified as aristolene epoxide by GC-MS. However, the specific production mechanism was unclear. The content change and the transformation rule of each component need to be further studied, and the key influencing factors should be clarified.

### Aflatoxins accumulation

The concentration of AFG_2_, AFG_1_, AFB_2_ and AFB_1_ showed a good linear relationship with the peak area. The matrix matching equations were *y* = 88500*x* − 19,000 (*r* = 0.9995), *y* = 40600*x* − 4470 (*r* = 0.9970), *y* = 48000*x* − 3240 (*r* = 0.9982), and *y* = 16400*x* − 8580 (*r* = 0.9987) for AFG_2_, AFG_1_, AFB_2_ and AFB_1_, respectively. The limits of quantification (LOQs) for AFG_2_, AFG_1_, AFB_2_ and AFB_1_ were 0.125, 0.1, 0.125 and 0.1 µg/kg, respectively.

**Fig. 5:**
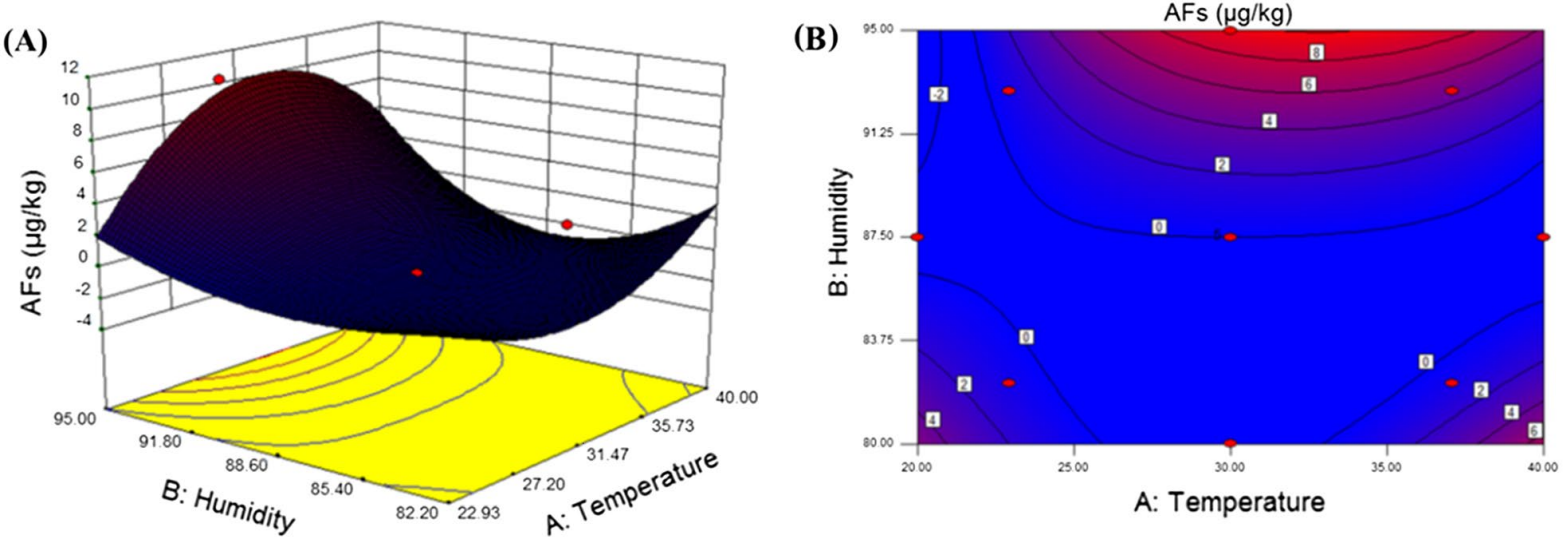
**A** Response surface plots, and **B** contours of various factors for *A. rhizoma*

The validated UFLC-MS/MS method applied practically to determine AFG_2_, AFG_1_, AFB_2_ and AFB_1_ in 14 batches of *A. rhizoma* sample before and after incubation with *Aspergillus flavus*. The highest concentration of total aflatoxins (AFG_1_ + AFG_2_ + AFB_1_ + AFB_2_) was 10.26 µg/kg in the sample that was incubated with *Aspergillus flavus* ans stored at 30 °C and 95% humidity. Also, 3.84 µg/kg of total aflatoxins was detected when the temperature and humidity were set at 37.07 °C and 92.80% humidity. These results were consistent with the mildew on the surface of samples and the content of atractylone, atractylenolide I, II, and III. Accordingly, response surface curve and contour plot in Fig. 5 were given according the experimental results in Table 2 based on CCD-RSM. It showed that the susceptible conditions for aflatoxins production in *A. rhizoma* was identified as temperature 22–37 °C and humidity over 87.5%.

### Exploration of suitable storage conditions for *A. rhizoma*

In this study, the characteristics, contents of moisture, chemical fingerprints changes and aflatoxins accumulation of *A. rhizoma* samples inoculation with *Aspergillus flavus* by artificial infection before and after different temperature and humidity condition were compared. The more serious the mildew of the sample was, the higher the content of aflatoxin in the sample was, and the more complex the change of chemical composition in the sample was. Aflatoxins accumulation seriously affected the chemical components in *A. rhizoma*, as well as its internal quality. Appropriate environmental conditions, especially environmental temperature and humidity, contributed to the growth of *Aspergillus flavus*, resulting in the changes in the content and kind of the bioactive components. It was preliminarily determined that the storage environment conditions suitable for the mildew prevention of *A. rhizoma* was set at lower than 22 °C and humidity less than 87.5%.

## Discussion

Aflatoxins are secondary metabolites produced by a wide variety of fungal species and constitute a significant hazard to the TCMs and food chains (Yang et al. 2017; Medina et al. 2017). Zhou et al. (2014) have illustrated that fungal contamination and mycotoxins residue will lead to the content reduction of the active components in TCMs, which might influence the inherent quality and safety of this TCM. Aflatoxins are unavoidable widespread natural contaminants of foods, feeds and TCMs with serious impacts on health, agricultural and livestock productivity, food and medicine safety (Noreddine 2020). The level of development of the country and the availability and degree of enforcement of pertaining regulations also account for the extent of TCMs and foods contamination with aflatoxins (Sirma et al. 2018). The poor level of development of the country exacerbates the risk and the extent of foods contamination due to faulty storage conditions that are usually suitable for mold growth and mycotoxin production: temperature of 22 to 29 °C and water activity of 0.90 to 0.99 (Noreddine 2020).

In this study, based on the trans-culture mode, CCD-RSM technique was used to establish a model to investigate the influence of environmental temperature and relative humidity on the growth of *Aspergillus flavus* and production of aflatoxins in *A. rhizoma* through the determination of the characteristics, contents of moisture, chemical fingerprints changes and aflatoxins accumulation of *A. rhizoma* samples before and after storage under different conditions. First, the color of *A. rhizoma* after storage had changed, and fungi can be clearly seen on samples. Second, the temperature and humidity had a certain effect on moisture content, and the value was higher than that of Chinese Pharmacopoeia (2020th Edition). Third, temperature had a greater effect on the type and quantity of chemical components than humidity. The contents of atractylone, atractylenolide I, II, and III changed most obviously. Also, a new component that aristolene epoxide identified by GC-MS appeared after a 10-day inoculation with *Aspergillus flavus*. But the reasons of this component need to be further studied. Lastly, aflatoxins were detected in the samples stored at 30 °C and 95% humidity, as well as 37.07 °C and 92.80% humidity. The results were consistent with the mildew on the surface of samples and the contents of atractylone, atractylenolide I, II, and III. It was found that the mildew of *A. rhizoma* was the most serious when it was stored in the temperature range of 22–37 °C and the humidity of 87.5%. This study provided a scientific basis for the rational storage and scientific maintenance of mildew prevention of *A. rhizoma* samples, ensuing the quality and safety of TCMs. Meanwhile, the methods should be applicable to TCMs that are susceptible to mycotoxigenic fungi contamination.

In view of the above-mentioned study, some suggestions were put forward for the safe storage of TCMs. First, the TCMs and relevant products should be stored in a ventilated, dry and cool place. The indoor temperature should be lower than 20 ^o^C, the relative humidity should be set at 45–75% to strictly control the water content of TCMs. It is forbidden to store TCMs at unqualified moisture. Secondly, the warehouse is equipped with cooling and dehumidification facilities, and electronic monitoring system to realize intelligent monitoring. Thirdly, the storage time of TCMs should be not too long. Fourthly, strengthening technical training for administrators to establish and improve a sound storage system for ensuring standardization, scientific and normalization of the storage of TCMs.

## Supplementary Information

**Additional file 1.** Fig. S1 Three-point inoculation and sporangia under optical microscope (Eyepiece 10×, Objective 400×) of *Aspergillus flavus*. Fig. S2 Response surface model of contents of atractylone, atractylenolide I, II and III.

## Data Availability

The data generated or analyzed during this study are included in this published article and its Additional file.

## Abbreviations

CCD-RSM: Central composite design-response surface methodology
TCMs: Traditional Chinese medicines
AFs: Aflatoxins
FAO: The Food and Agriculture Organization
WHO: The World Health Organization
UFLC-MS/MS: Ultra-fast liquid chromatography/tandem mass spectrometry
GC-FID: Gas chromatography with flame ionization detection
MgSO_4_: Magnesium sulfate anhydrous
SCDA: Czapek Dox Agar
ESI: Electrospray ionization
IS: Ion spray
CUR: Curtain gas
CAD: Collision activation dissociation gas
EP: Entrance potential
DP: Declustering potential
IS: Internal standard
RPA: Relative peak area
QuEChERS: Quick, Easy, Cheap, Effective, Rugged and Safe
LOQs: Limits of quantification
