# Supplementary material for: Physico-chemical characteristics and aflatoxins production of Atractylodis Rhizoma to different storage temperatures and humidities
Source: AMB Express. 2021 Nov 25;11:155. doi: 10.1186/s13568-021-01316-3 (PMC8617084; doi:10.1186/s13568-021-01316-3)
Supplement: Supplementary file 1 — Additional file 1. Fig. S1 Three-point inoculation and sporangia under optical microscope (Eyepiece 10×, Objective 400×) of Aspergillus flavus. Fig. S2 Response surface model of contents of atractylone, atractylenolide I, II and III. [file 13568_2021_1316_MOESM1_ESM.docx]

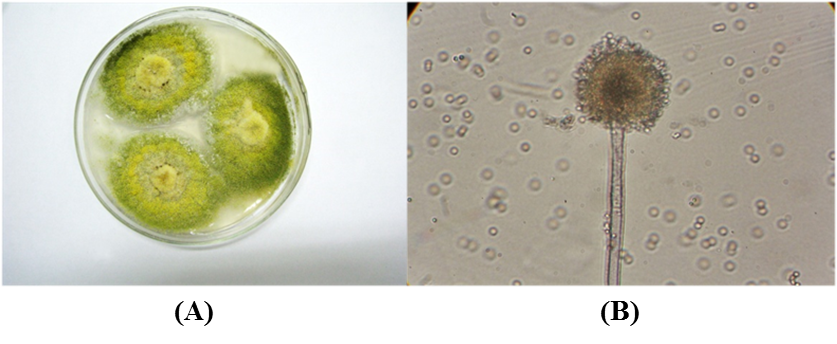


Fig. S1. Three-point inoculation and sporangia under optical microscope (Eyepiece 10×, Objective 400×) of *A. flavus*.


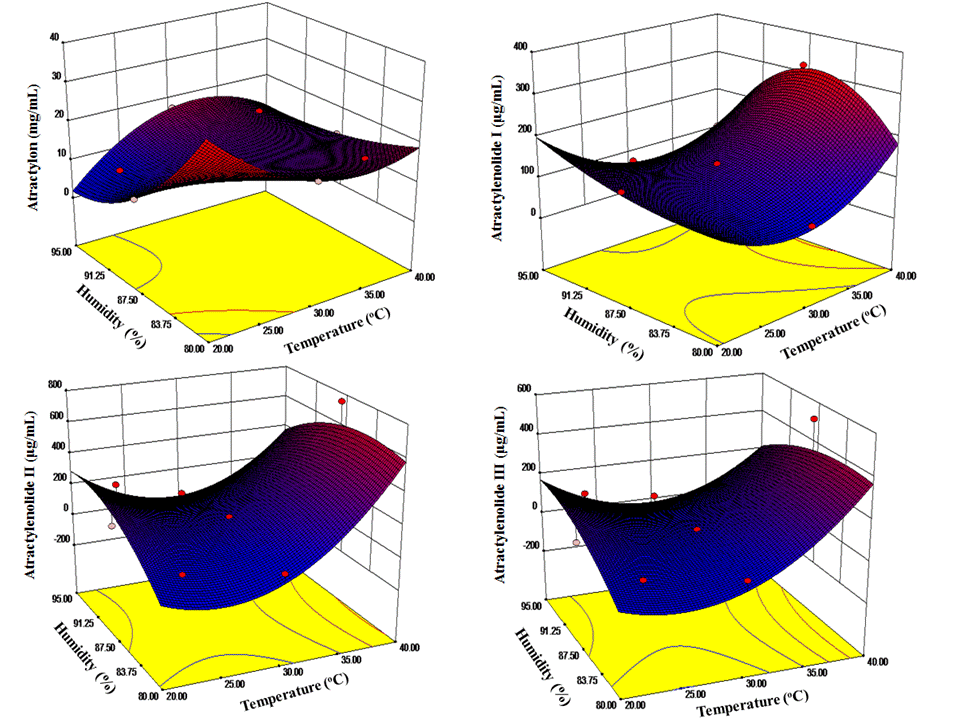


Fig. S2. Response surface model of contents of atractylone, atractylenolide I, II and III.
